# Serum Creatinine as a Potential Biomarker of Skeletal Muscle Atrophy in Non-small Cell Lung Cancer Patients

**DOI:** 10.3389/fphys.2021.625417

**Published:** 2021-04-12

**Authors:** Willian das Neves, Christiano R. R. Alves, Ana Paula de Souza Borges, Gilberto de Castro

**Affiliations:** ^1^Instituto do Cancer do Estado de São Paulo ICESP, Hospital das Clinicas HC FMUSP, Faculdade de Medicina da Universidade de São Paulo, São Paulo, Brazil; ^2^Center for Genomic Medicine, Massachusetts General Hospital, Boston, MA, United States

**Keywords:** cancer cachexia, muscle atrophy, creatinine metabolism, muscle wasting, sarcopenia

## Abstract

**Objectives:** Identifying simple biomarkers to determine muscle atrophy in non-small-cell lung cancer (NSCLC) patients remains a critical research gap. Since creatinine is mainly a product from intramuscular creatine metabolism, we tested the hypothesis that low serum creatinine levels would be associated to skeletal muscle atrophy in NSCLC patients.

**Materials and Methods:** This is a prospective cohort study including 106 treatment-naive patients with histologically confirmed stage IV NSCLC. All patients performed routine serum creatinine laboratory tests. We divided patients into two groups based on low (<0.7 mg/dL for male and <0.5 mg/dL for female) or normal creatinine levels. We compared body mass index (BMI), psoas muscle cross-sectional area, adipose tissue area and complete blood counts between groups.

**Results:** Male and female NSCLC patients with low serum creatinine levels had low muscle cross-sectional area as compared to patients with normal serum creatinine levels. Male NSCLC patients with low serum creatinine also displayed reduced BMI, reduced adipose tissue area, and elevated systemic inflammation compared to NSCLC patients with normal serum creatinine levels. There were no significant differences between female groups for BMI, adipose tissue area and inflammatory markers.

**Conclusions:** Serum creatinine is a potential prognostic biomarker of skeletal muscle atrophy in NSCLC patients. Since serum creatinine is a simple and accessible measurement, we suggest that it should be monitored in longitudinal follow-up of NSCLC patients as a biomarker of muscle atrophy.

## Introduction

Non-small-cell lung cancer (NSCLC) patients are usually diagnosed in advanced stages and frequently present severe weight loss. At the time of the diagnostic, about half of the NSCLC patients have cancer cachexia, a syndrome characterized by loss of muscle mass, with or without loss of adipose tissue (Fearon et al., 2011). Cancer cachexia is associated with a poor prognosis, including decreased response to therapies (Fearon et al., 2011). Measurement of muscle mass in NSCLC patients are frequently performed using computed tomography (CT) scans, which is time-consuming and demands imaging analysis (Nishimura et al., 2019). Identifying accessible biomarkers to determine and track skeletal muscle atrophy in NSCLC patients remains a major research gap.

Creatine is a nitrogenous amine that plays a crucial bioenergetic role in tissues with high metabolic demand. Over 90% of the total creatine is stored in skeletal muscles and it is converted into creatinine, resulting in turnover of about 1.7% of the total creatine/day. Elevated serum creatinine levels are the most used biomarker to indicate impaired kidney function in the clinical setting. However, as serum creatinine is mainly a waste product from intramuscular creatine metabolism, conditions involving skeletal muscle wasting often show low serum creatinine levels as previous reported in patients with chronic kidney disease (Patel et al., 2013), Duchenne muscular dystrophy (Wang et al., 2017), amyotrophic lateral sclerosis (van Eijk et al., 2018), and spinal muscular atrophy (Alves et al., 2020). Potential associations between low serum creatinine levels and skeletal muscle atrophy have not been previously investigated in NSCLC patients.

As serum creatinine analyses are conducted as the routine work-up in NSCLC patients, we hypothesized that it could be a simple and an accessible biomarker to determine skeletal muscle atrophy in NSCLC patients. Thus, the aim of this study was to evaluate whether low serum creatinine levels would be associated with lower muscle cross sectional area in NSCLC patients when compared to patients with normal serum creatinine levels. We split a cohort of NSCLC patients into two groups based on low or normal serum creatinine levels, and assessed body mass index (BMI), psoas muscle cross-sectional area and adipose tissue area as main endpoints. We also tested platelet to lymphocyte and neutrophil to lymphocyte ratios in peripheral blood cell counts as inflammation markers.

## Methods

This is a prospective cohort study approved by the local ethical committee of the Faculty of Medicine of University of São Paulo (CEP 802/15 and 1105/17; clinicaltrial.gov NCT03960034 and NTC04306094) all studied patients was included between April 2017 and May 2020, to characterize cachexia and muscle atrophy in advanced NSCLC and their prognostic impact. The present analysis includes a total of 106 treatment-naive patients with histologically confirmed stage IV NSCLC. Clinical characteristics were collected at study admission (signed informed consent) and included age, sex, anthropometric data, histologic type and ECOG performance status. All patients performed routine laboratory tests including assessment of serum creatinine (measured by Jaffe colorimetric method), and complete blood count. For statistical analysis, we split patients into two groups based on low (<0.7 mg/dL for males and <0.5 mg/dL for females) or normal creatinine levels (0.7–1.5 mg/dL for males and 0.5–1.0 mg/dL for females) (Pottel et al., 2008). Analysis was performed independently for male and female patients. BMI, psoas muscle area, adipose tissue area and cell blood counts were compared between groups. To determine psoas muscle area and adipose tissue area, plain computed tomography images were checked at the level of the third lumbar vertebra and used to calculate the psoas cross-sectional area and adipose tissue area (SliceOmatic, TomoVision, Magog, Canada; http://www.tomovision.com). Briefly, tissues were semi−automatically selected with the Alberta Protocol. Tissues were selected using radiological densities between default ranges, where −29 to 150 hounsfield units (HU) were used for skeletal muscle, −150 to −50 HU for visceral adipose tissue, and −190 to −30 HU for intermuscular and subcutaneous adipose. Since we are studying NSCLC patients, all subjects have available tomography acquired during the screening for lung cancer and/or clinical staging (Postmus et al., 2017). Psoas muscle CSA was easily quantified using these images and previous studies demonstrated that psoas mass is associated with whole body muscle mass (Shen et al., 2004) and the specific region where psoas is located is better quantified using these scans than other regions (Derstine et al., 2018).

Results are reported as means ± standard deviation (SD). Shapiro-Wilk test was applied to test normality. Unpaired two-tailed *t*-test was performed to compare groups for variable with normal distribution, while Mann Whitney test was performed to compare groups for variables with non-normal distribution. The level of significance was defined as *p* < 0.05.

## Results

**FIGURE 1:**
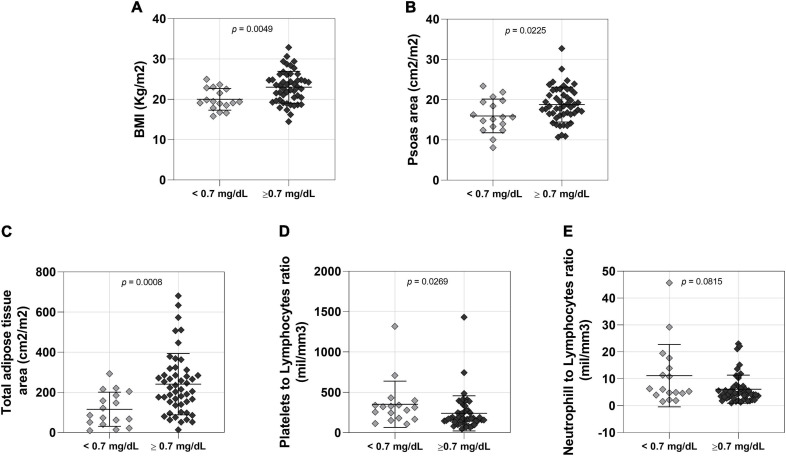
Comparison between lower (*n* = 17 < 0.7 mg/dL) and normal (*n* = 49 > 0.7 mg/dL) creatinine level male patients. **(A)** BMI: body mass index; <0.7 mg/dL 57.53 ± 9.234 (mean ± SD), >0.7 mg/dL 67.37 ± 13.03 (mean ± SD); 95% CI 0.9478–5.077. **(B)** *Psoas area*; <0.7 mg/dL 15.96 ± 4.187 (mean ± SD), >0.7 mg/dL 18.79 ± 4.340 (mean ± SD); 95% CI 0.4132–5.2520. **(C)** *Total adipose tissue*; <0.7 mg/dL 116.0 ± 85.25 (mean ± SD), >0.7 mg/dL 240.9 ± 152.5 (mean ± SD); 95% CI 43.50–172.6. **(D)** *Platelets to lymphocytes ratio*; <0.7 mg/dL 353.2 ± 286.7 (mean ± SD), >0.7 mg/dL 240.4 ± 217.9 (mean ± SD); 95% CI –164.9 to –7.320 **(E)** *Neutrophill to lymphocytes ratio*; <0.7 mg/dL 11.17 ± 11.60 (mean ± SD), >0.7 mg/dL 6.132 ± 5.229 (mean ± SD); 95% CI –6.080 to 0.2500.

Baseline NSCLC patients’ characteristics are presented in Table 1. This cohort included 66 male patients and 40 female patients. Male patients with low serum creatinine levels had lower body weight (Supplementary Table 1), BMI (Figure 1A), psoas muscle cross-sectional area (Figure 1B) and adipose tissue (Figure 1C and Supplementary Table 1) when compared to male patients with normal serum creatinine levels. Moreover, male patients with low creatinine had lower hemoglobin and hematocrits levels, with no differences in the other blood cell counts (Supplementary Table 1). We observed higher platelet to lymphocyte (Figure 1D) and neutrophil to lymphocyte (Figure 1E) ratios in patients with low creatinine levels, which are inflammatory markers.

**TABLE 1:** Baseline characteristics of the study population.

Total (*N* = 106)	Mean ± SD	
Age	63.40 ± 9.6	
Body weight (kg)	65.1 ± 14.2	
BMI	22.5 ± 4.5	
	**N**	**%**
**Sex**		
Male	66	62.3
Female	40	37.7
**ECOG PS Status**		
0	7	6.6
1	16	15.1
2	53	50.0
3	23	21.7
4	7	6.6
**Histologic type**		
Adenocarcinoma	70	66.0
CEC	28	26.4
NSCLC-NOS	8	7.6
**Weigth loss > 5% in the last 6 months**		
Yes	86	81.1
No	20	18.9

**FIGURE 2:**
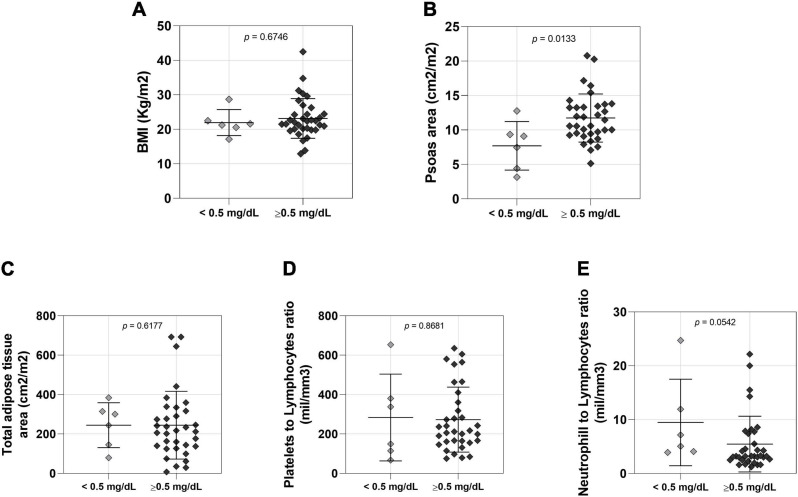
Comparison between lower (*n* = 6 < 0.5mg/dL) and normal (*n* = 34 > 0.5mg/dL) creatinine level female patients. **(A)** BMI: body mass index; <0.5 mg/dL 21.95 ± 3.768 (mean ± SD), >0.5 mg/dL 23.16 ± 5.749 (mean ± SD); 95% CI –2.430 to 4.510. **(B)** Psoas area; <0.5 mg/dL 7.704 ± 3.517 (mean ± SD), >0.5 mg/dL 11.73 ± 3.496 (mean ± SD); 95% CI 0.8896 to 7.163. **(C)** Total adipose tissue; <0.5 mg/dL 244.2 ± 113.8 (mean ± SD), >0.5 mg/dL 244.4 ± 171.8 (mean ± SD); 95% CI –141.8 to 97.40. **(D)** Platelets to lymphocytes ratio; <0.5 mg/dL 283.5 ± 220.2 (mean ± SD), >0.5 mg/dL 272.6 ± 165.1 (mean ± SD); 95% CI –181.3 to 144.8. **(E)** Neutrophill to lymphocytes ratio; <0.5 mg/dL 9.480 ± 8.036 (mean ± SD), >0.5 mg/dL 5.470 ± 5.174 (mean ± SD); 95% CI –8.700 to 0.0800.

Female NSCLC patients were also studied into two groups based on low and normal serum creatinine levels. We did not find significant differences in body mass (Supplementary Table 2) and BMI (Figure 2A) between these two groups, but patients with low serum creatinine levels had reduced muscle cross-sectional area compared to patients with normal serum creatinine (Figure 2B). There was no statistically significant difference between female groups in adipose tissue area (Figure 2C and Supplementary Table 2), platelet to lymphocyte (Figure 2D) and neutrophil to lymphocyte ratios (Figure 2E).

All male and female patients analyzed in this study with ECOG PS status 0 had normal creatinine levels. Altogether, these data indicate that male and female NSCLC patients with low serum creatinine levels have lower muscle mass.

## Discussion

Cancer cachexia is associated with a poor prognosis and identifying better prognostic biomarkers to determine the onset of muscle atrophy and to further track muscle atrophy in cancer patients remains a major research gap for researchers and clinicians. In addition to the obvious measurement of body weight, different circulating factors have been proposed as candidates to refine the diagnoses of cancer cachexia (Fearon et al., 2011). Here, we demonstrate that serum creatinine is a potential prognostic biomarker of skeletal muscle atrophy in NSCLC patients. Remarkably, low levels of creatinine are also associated to systemic inflammation, a hallmark of cancer cachexia progression (Argilés et al., 2019).

Many authors have already reported that low serum creatinine concentrations could be associated to changes in skeletal muscle mass and cumulative data denote low levels of serum creatinine in different diseases involving muscle atrophy (Patel et al., 2013; Wang et al., 2017; van Eijk et al., 2018; Alves et al., 2020). Importantly, as elevated serum creatinine is an established marker of impaired kidney function, most physicians commonly request this laboratory test to monitor renal function, which makes it a very accessible measurement. However, we also speculate that low levels of serum creatinine are often not emphasized as an alarming sick condition in the clinical practice. Corroborating with our findings, Thongprayoon et al. (2017) evaluated the association between the admission creatinine value and in-hospital mortality in a total of 73,994 different type of patients and showed that low serum creatinine concentrations at admission were associated with greater in-hospital mortality (Thongprayoon et al., 2017). Moreover, low levels of creatinine are associated with higher mortality rate in melanoma patients as demonstrated in a retrospective cohort study where low serum creatinine levels were associated with worse survival in 139 melanoma cancer patients treated with anti-PDL-1 and anti-CTL4 (Naik et al., 2019). Altogether, these findings highlight the importance of interpreting low levels of serum creatinine as a candidate biomarker of both skeletal muscle atrophy and poor prognosis in cancer patients.

We could analyze data available from two independent studies (clinicaltrial.gov NCT03960034 and NTC04306094), including 66 male and 40 female patients. One explanation for the lack of significant differences in female patients on inflammation could be related to the studied number of patients, which is a limitation of this study. There is a possibility that we indeed have sex differences during cancer progression, with serum creatinine being able to determine loss of adipose tissue mass in males, but not female patients. In this sense, sex differences in skeletal muscle and adipose tissue physiology have being extensively reviewed and should be considered to discuss our findings (Montalvo et al., 2018; Chang et al., 2018). In addition, another limitation to be considered is the sensitive of serum creatinine for the severe cases of muscle atrophy. The lowest value detectable and observed in a patient is 0.2 mg/dL measured by Jaffe colorimetric method. However, we believe that for those severe cases with extreme low levels, the muscle atrophy condition would be easily confirmed by clinical examination as well as potential treatment responses.

Since serum creatinine is a very accessible measurement, moving forward we can consider it as a surrogate marker for imaging analysis in the clinical setting. Moreover, these findings provide foundation to test serum creatinine levels in other populations with high prevalence of cancer cachexia. We certainly encourage a validation of the current data in additional cohorts, including longitudinal cohort studies, and different cancer types to refine the criteria applied to determine muscle atrophy in cancer patients. Of note, future analysis of urine levels of creatinine would be also interested to understand if similar findings can be observed in urine samples. Finally, future studies are necessary to address the prognostic impact of low creatinine levels on overall survival in cancer patients.

## Data Availability Statement

The original contributions presented in the study are included in the article/Supplementary Material, further inquiries can be directed to the corresponding author/s.

## Ethics Statement

The studies involving human participants were reviewed and approved by USP – Faculdade de Medicina da Universidade de São Paulo – FMUSP. The patients/participants provided their written informed consent to participate in this study.

## Author Contributions

WN, CA, and GC conceived and designed the study. WN and AS conducted patient’s inclusion procedures. WN and CA performed data analysis and drafted the manuscript. All authors have participated in the manuscript review and approved the final manuscript.

## Conflict of Interest

The authors declare that the research was conducted in the absence of any commercial or financial relationships that could be construed as a potential conflict of interest.
