# Supplementary material for: Serum Creatinine as a Potential Biomarker of Skeletal Muscle Atrophy in Non-small Cell Lung Cancer Patients
Source: Front Physiol. 2021 Apr 12;12:625417. doi: 10.3389/fphys.2021.625417 (PMC8072336; doi:10.3389/fphys.2021.625417)
Supplement: Supplementary file 1 [file Table_1.docx]

**Supplementary Table 1.** Body composition and blood cell count according to serum creatinine levels in male patients (N=66).

|  | **Crn < 0.70** | **Crn ≥ 0.70** | **N = 66** | |
| --- | --- | --- | --- | --- |
|  | Mean ± SD | Mean ± SD | p value | 95% CI |
| Body composition | | | | |
| Body weigth (kg) | 57.53 ± 9.234 | 67.37 ± 13.03 | 0.0056 | 2.983 to 16.69 |
| VAT (cm^2^/cm^2^) | 57.03 ± 48.10 | 124.0 ± 92.80 | 0.0020 | 17.56 to 94.80 |
| SAT (cm^2^/cm^2^) | 51.18 ± 36.98 | 104.3 ± 65.41 | 0.0007 | 19.05 to 74.12 |
| IMAT (cm^2^/cm^2^) | 6.644 ± 6.644 | 12.58 ± 8.587 | 0.0239 | 0.5600 to 8.228 |
| Cell blood counts | | | | |
| Hemoglonin (g/dl) | 11.08 ± 2.331 | 20.19 ± 29.21 | 0.0030 | 0.8000 to 3.700 |
| Hematocrit (%) | 34.18 ± 6.497 | 38.90 ± 5.431 | 0.0046 | 1.511 to 7.940 |
| Leucocytes (mil/mm3) | 13.02 ± 6.707 | 10.95 ± 3.708 | 0.6735 | -3.020 to 1.400 |
| Neutrophill (mil/mm3) | 10.66 ± 6.846 | 8.002 ± 3.758 | 0.2477 | -3.900 to 1.020 |
| Linphocytes (mil/mm3) | 1.387 ± 0.6478 | 1.765 ± 0.9761 | 0.1425 | -0.100 to 0.640 |
| Platelets (mil/mm3) | 376.3 ± 157.9 | 319.7 ± 121.2 | 0.1217 | -104.0 to 14.00 |
| VAT = Visceral adipose tissue; SAT = subcutaneous adipose tissue; IMAT = intramuscular adipose tissue. | | | | |

**Supplementary Table 2.** Body composition blood cell count according to serum creatinine levels in female

patients (N=40)

|  | **Crn < 0.50** | | **Crn ≥ 0.50** | | | **N= 40** | |  |
| --- | --- | --- | --- | --- | --- | --- | --- | --- |
|  | Mean ± SD | | | | Mean ± SD | p value | 95% CI | |
| Body composition | | | | | | | | |
| Body weigth (kg) | | 53.17 ± 4.262 | | 56.38 ± 15.94 | | 0.9191 | -8.000 to 9.000 | |
| VAT (cm2/cm2) | | 89.01 ± 59.86 | | 71.76 ± 48.52 | | 0.4422 | -62.21 to 27.71 | |
| SAT (cm2/cm2) | | 135.2 ± 54.35 | | 157.1 ± 123.9 | | 0.9559 | -60.90 to 75.80 | |
| IMAT (cm2/cm2) | | 19.97 ± 18.47 | | 15.57 ± 11.33 | | 0.6441 | -10.70 to 7.510 | |
| Cell blood counts | | | | | | | | |
| Hemoglonin (g/dl) | | 10.20 ± 2.016 | | | 11.52 ± 1.590 | 0.0791 | -0.1608 to 2.802 | |
| Hematocrit (%) | | 30.72 ± 6.106 | | | 35.71 ± 4.423 | 0.0209 | 0.7977 to 9.187 | |
| Leucocytes (mil/mm3) | | 14.69 ± 4.090 | | | 9.244 ± 2.979 | 0.0004 | -8.266 to -2.623 | |
| Neutrophill (mil/mm3) | | 11.83 ± 3.340 | | | 6.582 ± 2.841 | 0.0038 | -8.180 to -2.400 | |
| Linphocytes (mil/mm3) | | 1.773 ± 1.040 | | | 1.726 ± 0.9405 | 0.8621 | -1.030 to 0.8200 | |
| Platelets (mil/mm3) | | 336.5 ± 98.67 | | | 362.5 ± 117.5 | 0.6127 | -77.21 to 129.3 | |
| VAT = Visceral adipose tissue; SAT = subcutaneous adipose tissue; IMAT = intramuscular adipose tissue. | | | | | | | |  |
